# High-Speed Edge Trimming of CFRP and Online Monitoring of Performance of Router Tools Using Acoustic Emission

**DOI:** 10.3390/ma9100798

**Published:** 2016-09-26

**Authors:** Rangasamy Prakash, Vijayan Krishnaraj, Redouane Zitoune, Jamal Sheikh-Ahmad

**Affiliations:** 1 PSG College of Technology, Coimbatore 641004, India; rprakash.psg@gmail.com; 2 University Paul Sabatier, Toulouse 31300, France; redouane.zitoune@iut-tlse3.fr; 3 The Petroleum Institute, P.O. Box 2533, Abu Dhabi, United Arab Emirates; jahmad@pi.ac.ae

**Keywords:** CFRP, trimming, router tools, force, delamination, acoustic emission, ANN

## Abstract

Carbon fiber reinforced polymers (CFRPs) have found wide-ranging applications in numerous industrial fields such as aerospace, automotive, and shipping industries due to their excellent mechanical properties that lead to enhanced functional performance. In this paper, an experimental study on edge trimming of CFRP was done with various cutting conditions and different geometry of tools such as helical-, fluted-, and burr-type tools. The investigation involves the measurement of cutting forces for the different machining conditions and its effect on the surface quality of the trimmed edges. The modern cutting tools (router tools or burr tools) selected for machining CFRPs, have complex geometries in cutting edges and surfaces, and therefore a traditional method of direct tool wear evaluation is not applicable. An acoustic emission (AE) sensing was employed for on-line monitoring of the performance of router tools to determine the relationship between AE signal and length of machining for different kinds of geometry of tools. The investigation showed that the router tool with a flat cutting edge has better performance by generating lower cutting force and better surface finish with no delamination on trimmed edges. The mathematical modeling for the prediction of cutting forces was also done using Artificial Neural Network and Regression Analysis.

## 1. Introduction

Carbon Fiber reinforced polymers have been extensively used in aerospace, transportation, robotics, sporting goods, construction, medical, and military applications due to its high strength-to-weight ratio and high modulus-to-weight ratio [1]. CFRP composites contain two phases of materials with significantly distinguished mechanical and thermal properties, causing complex interactions between the matrix and the reinforcement during machining. In CFRP composites, the reinforcement is carbon fiber [2] and the matrix is usually a polymer resin—such as epoxy—which provides the strength to bind the reinforcements together. Even though composite components are often made to near-net shapes, after demolding some post-machining operations are often unavoidable, like drilling and trimming. These operations must be performed to assure that the composite parts meet dimensional tolerance, surface quality, and other functional requirements [3]. There is a substantial difference between the machining of metals, their alloys, and that of composite materials because of their anisotropy and inhomogeneity [4]. CFRP composites pose significant problems in milling such as fiber pull-out, delamination, fuzzing, and thermal degradation [5]. Machining of a composite materials is difficult to carry out due to their mechanical and thermal properties—heterogeneity, anisotropy, and low thermal conductivity—and to the high abrasiveness of their reinforcing constituents. These properties typically result in damage being introduced into the work material and in very rapid wear development in the cutting tool [6]. Teti [7] described that the fiber type, the reinforcement construction, and the matrix content are the most important factors governing selection of cutting tool and machining parameters. Masahiro Hagino et al. [8] found that the fiber orientation is another critical factor which significantly influence the tool wear while machining CFRPs with milling tool. The conventional post-machining processes which are essentially to be carried out after demolding CFRP components are edge trimming or routing, milling, drilling, countersinking, and grinding [9,10,11]. A variety of cutting tool materials and geometries are available for machining FRPs. One reason for this variety in tooling is the multiple characteristics of the FRP products deriving from the various forms, types of reinforcement, matrices, and volume fraction of reinforcement fibers that are utilized for various applications. Haddad Madjid [12] investigated the influence of cutting parameters and tool wear on the machined surface quality during trimming of multidirectional CFRP laminates with a burr tool. The defects in the free edges are mainly influenced by the machining parameters (such as speed, feed, depth of cut, and fiber orientation) and the type of machining (orthogonal cutting and oblique cutting) [13]. Jamal Sheikh-Ahmad [14] discussed that the fluted tools generate an axial force, which acts normal to the stacking direction of the laminate. These are cutting forces in turn cause defects such as delamination and fuzzing in the surface plies [15,16,17,18]. The measurement of resultant cutting force is essential since more cutting forces are not favorable as they damage the CFRP material [1,19]. Therefore, it is necessary to predict the cutting forces for selecting process parameters that would result in minimum machining damage in edge trimming [20,21]. Because of the non-homogeneous nature of composite materials, their response to machining may involve undesirable consequences such as rapid tool wear, fiber pullout, and delamination. All of these reactions are directly related to the cutting tool forces generated on the workpiece edge. Delamination, in particular, is strongly dependent on the cutting force normal to the stacking plane in composites. In edge trimming, the delamination is caused by the tensile axial cutting force component. Sreenivasulu [22] quantified the surface quality based on delamination depth and surface roughness, found that delamination depth and surface roughness increase with an increase in feed and an increase in cutting distance, and decrease with an increase in spindle speed. Tool wear and failure monitoring have raised a lot of interest among researchers, as it can help to prevent damage of workpieces as well as improve the quality of the surface finish. In principle, tool wear monitoring methods can be classified into two categories, namely direct and indirect methods. Direct measurement of tool performance using optical methods requires that the machine be stopped, and the tool has to be removed from the spindle and visually inspected which results in machine downtime and human intervention costs. Indirect methods of tool performance monitoring without shutting down the machine can be realized without interrupting the production process and can be performed by signal collection and processing. Measurement of tool wear is also problematic in cases of complex cutting tool geometries, such as router tools and abrasive cutters. In these cutting tools, multiple faces and cutting edges are engaged in the cut and wear is often not uniform. It is found to be more reasonable in such cases that tool wear is measured indirectly and online by monitoring the cutting forces, using acoustic emission (AE) signals [23,24,25]. Though many methods exist for tool wear monitoring, the AE signal is very effective for indirect methods because of ease of operation and fast dynamic response. Elastic stress waves (AE waves) produced by the release of strain energy during machining (deformation and fracture of materials) are used in nondestructive inspection and analysis. These elastic stress waves are typically measured employing an AE sensor that converts them into an electrical voltage signal. Many studies have been done on monitoring the state of machining surfaces and the condition of tools by the analysis of the associated acoustic emissions (AE) [26,27]. AE mean value changes in response to wear of the cutting tool and the roughness of the machined surface. The formation of chips, breakage and collision of chips, rubbing action between the cutting tool and chips, rubbing action between the cutting tool and the work piece, and failure and wear of the cutting tool are the primary sources of AE waves [28,29,30]. The usefulness of the AE method in detecting damage on CFRP trimmed edges (delamination and Surface roughness) and cutting tools (breakage, chipping, etc.) has been promoted to investigate on edge trimming on CFRP materials using router tools [31]. Prakash [32] has found a strong relationship between tool wear and acoustic emission signals and surface roughness (*Ra*) in micro end milling. Therefore, in this study, cutting experiments involving various cutting conditions and router cutting tools were performed to investigate the relationship between AE signals and the state of a cutting operation regarding tool wear and the roughness of the trimmed surface [33]. Although Artificial Neural Network is ideally suited for predicting complex fiber reinforced problems because it can be trained easily to find solutions. Kalla et al. [34,35] showed that Artificial Neural Networks had a lot of potential to offer for application in the modeling of fiber reinforced polymers machining processes. The limited amount of published literature on the fundamentals of CFRP machining, especially with router tools, motivated to conduct a series of experiments for correlating the cutting force, tool performance, and surface roughness obtained with different cutting conditions and tool geometries [36]. This work focuses on selecting the suitable tool for edge trimming of CFRP. AE method has been used for evaluating the ease of machining of router tools in edge trimming.

## 2. Materials and Methods

### 2.1. Workpiece Material

**Figure 1:**
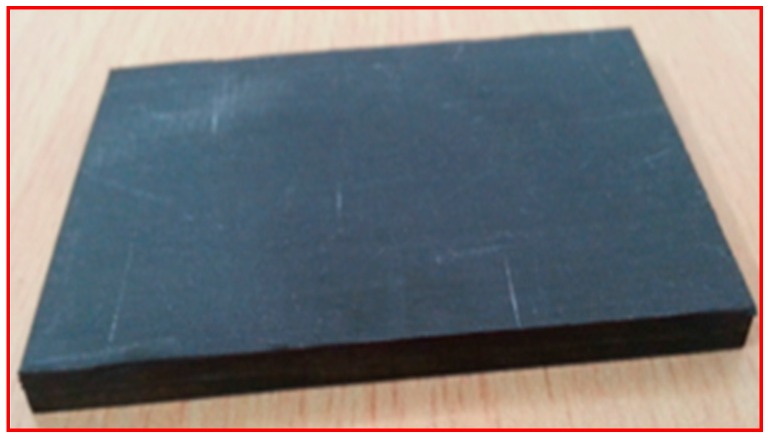
CFRP Specimen.

Carbon fiber reinforced polymer (CFRP) composite of 4.16 mm thickness (16 layers, each of 0.26 mm thick) was used for conducting the trimming studies. The CFRP was made using unidirectional prepregs supplied by Hexcel Composite Company referenced under HEXPLY UD T700 268 M21 34% (T700-M21). The stacking sequence of the laminate [90/−45/0/45/90/−45/0/45]_s_ so as to get a multidirectional laminate. CFRP laminates were compacted using a vacuum pump and then cured in an autoclave. The nominal fiber volume fraction was found to be 0.59. The size of the specimen for the experimentation was taken as 40 × 70 × 4.16 mm. Figure 1 shows the sample of CFRP material used in the investigation.

### 2.2. Cutting Tools

CFRP materials are highly abrasive in nature. Machining of CFRP involves both the cutting and shearing action of fibers to be occurring simultaneously. Hence, cutting tools should incorporate a unique tool geometry that effectively responds to these requirements. This study was conducted with two variety of burr tools with different geometries and one normal fluted tool to understand the performance on trimming and also to evaluate the quality of trimmed edges.

**Figure 2:**
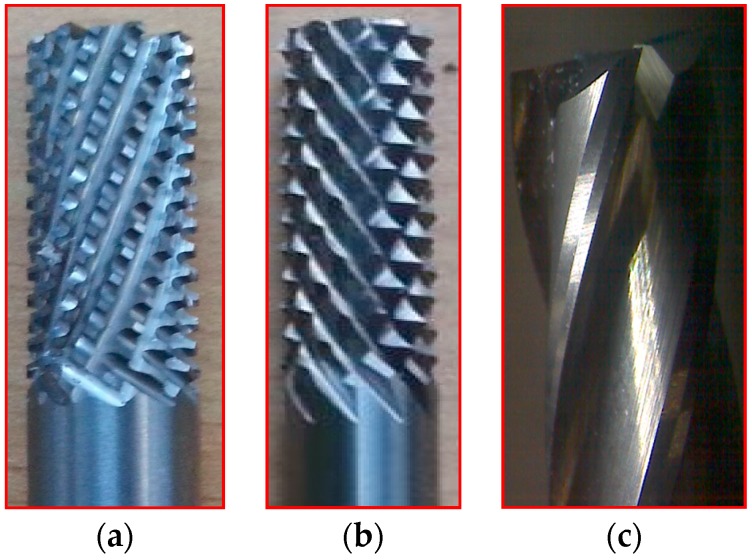
(**a**) Tool T1 (**b**) Tool T2 (**c**) Tool T3.

Three tools of Ø 6 mm, made up of tungsten carbide—namely router type (T1), router type (T2) and four fluted helical end mill (T3) as shown in Figure 2a–c—were selected. Table 1 shows the detailed specification of three different tools used in edge trimming of CFRP material. The experimental investigation was made under dry conditions.

### 2.3. Experimental Setup

**Figure 3:**
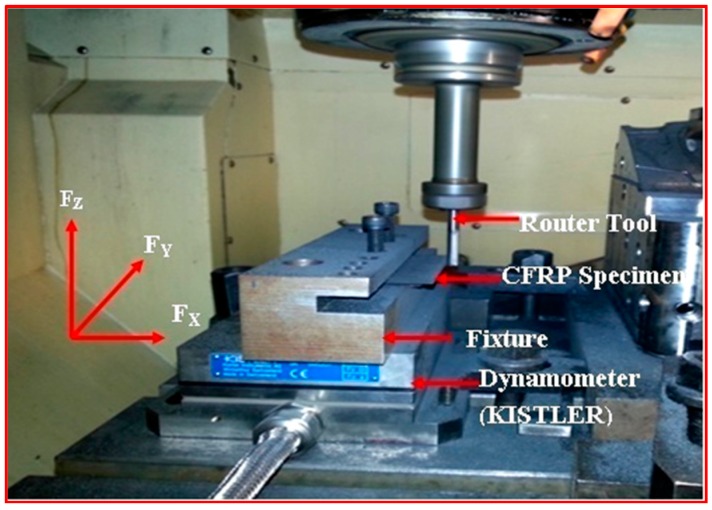
Edge trimming.

After demoulding of CFRP parts, they need to be trimmed at the edges for meeting the desired surface integrities. Such a process is called edge trimming. Edge trimming is also called peripheral milling because the tool diameter is usually small and the axial engagement encompasses the entire thickness of the workpiece. Figure 3 shows the process of edge trimming (up-milling).

Edge trimming trials were performed on high spindle speed Makino S33 vertical machining center (VMC). Table 2 shows the specification of the machining center used in CFRP machining.

A Kistler type 9257BA tri-axis piezoelectric dynamometer was used for measuring the cutting forces in all three directions—namely the feed direction (*F_y_*), normal to the feed (*F_x_*), and the axial direction (*F_z_*). The dynamometer consists of four three-component force sensors fitted under high preload between a base plate and a cover plate. A three-channel charge amplifier is built-in the dynamometer. Therefore, the output signal at the dynamometer is of low impedance. The integrated cable is connected to the control unit Type 5233A1. The control unit can select the four measuring ranges in two groups (*F_x_* and *F_y_* resp. *F_z_*). The control unit is easy to operate and contains power pack and keyboard with status displays together with a connector for signal input. The output voltages are proportional to the forces occurring. The cutting forces were acquired and recorded using Dynoware software.

**Figure 4:**
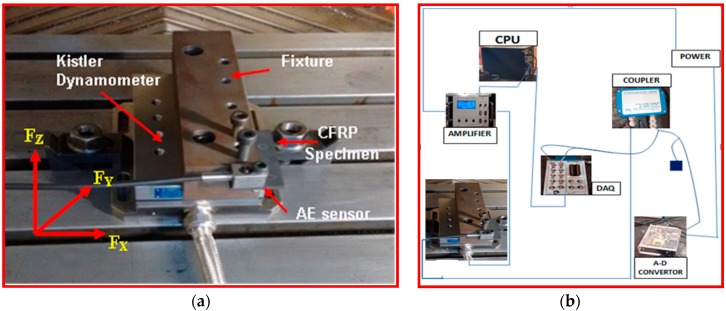
(**a**) Experimental setup for Cutting force and Acoustic Emission Measurement; (**b**) Wiring diagram of devices connected to measure forces and performance of cutting tool.

A Kistler type (8152B111) AE sensor with a frequency range from 50 to 400 kHz with a sampling rate frequency of 2.5 Ms/s (Mega samples per second), i.e., 2.5 MHz was bolted in the CFRP specimen. The AE Piezotron Coupler Type 5127B1 is used to supply power to the sensor and for signal processing. The signals from the sensor were then fed into a coupler to process high-frequency sound emission signals. The output signals from the AE sensor were amplified to a level of 60 dB gain by using an amplifier. The amplifier has two series-connected second order filters designed as plug-in elements. A band pass filter is obtained by the series connection of one high-pass and one low-pass filter to eliminate noise and signals caused by phenomena such as collisions and twining of chips that are not directly related to the cutting phenomenon [33]. The data acquisition system was used to acquire signals from the coupler. The AE signals were mainly evaluated by using an analog output signals parameter AE Out (Filter) mean value in volts (which is one of the outputs of AE signal from the coupler like AE rms signal. AE signal obtained from the signal processing corresponding to fluctuations in the amplitude of the AE signals [37]. Figure 4a shows the setup for measurement of cutting forces and acoustic emission signals. Figure 4b shows the wiring diagram of devices connected to measure force and performance of cutting tool. Mitutoyo makes a surface roughness tester (model SJ-411), and Tool maker’s microscope was used to study the state of machined surfaces such as Surface roughness (*Ra*) and delamination respectively. Measurement of cutting forces (*F_x_*, *F_y_* and *F_z_*) and surface roughness were done with three trials on each condition. Then the mean values were calculated for analysis.

Experiments were conducted using the full factorial design of L9 orthogonal array. The cutting conditions were selected to generate different level of mechanical degradation. Table 3 shows the summary of machining conditions and the different levels. The axial depth of cut was equal to the laminate thickness of 4.16 mm.

## 3. Results and Discussion

### 3.1. Cutting Force

The cutting force generated during the edge trimming process due to various cutting parameters and tool geometries was measured using the Kistler milling tool dynamometer. Three trials were done on each combination of cutting conditions in calculating the resultant force *R*. The Equation (1) is used for calculating the resultant cutting forces.

(1) $$R(N)=\sqrt{{\left(F_{x}\right)}^{2}+{\left(F_{y}\right)}^{2}+{\left(F_{z}\right)}^{2}}$$

**Figure 5:**
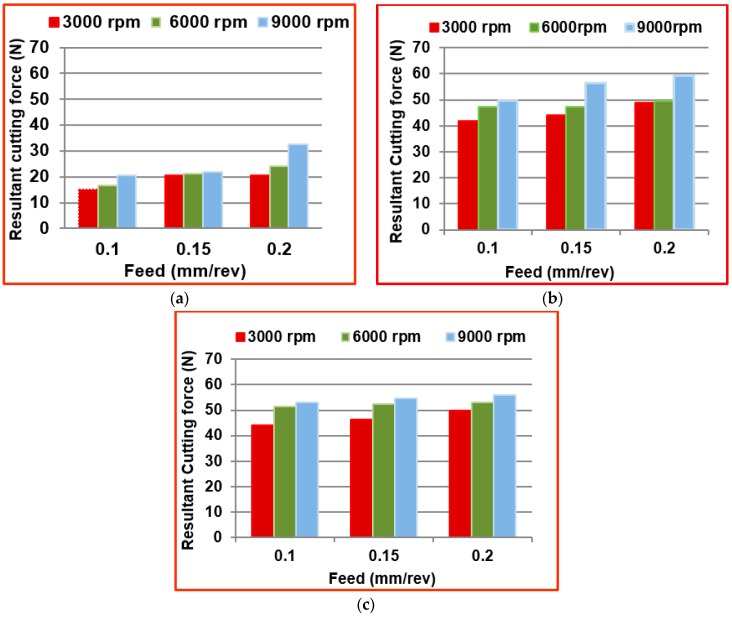
Comparison of resultant cutting forces at different machining conditions with (**a**) Tool T1; (**b**) Tool T2; and (**c**) Tool T3.

Table 4 shows the calculated resultant cutting forces *R(N)* and measured surface roughness (*Ra* in µm) values at the different conditions while edge trimming using the three different tools namely T1, T2, and T3. Figure 5a–c shows the effect of various tool geometries and cutting parameters on the resultant cutting forces.

From the Figure 5, it was observed that the resultant cutting force increases with increase in spindle speed as well as feed for all the three kinds of tools. By considering all the combination of machining parameters (Spindle speed and feed) in this study, the tool T1 has generated lower cutting force during machining than other two tools because of the trapezoidal shape of the cutting teeth. The tool T2 has generated comparatively higher cutting forces because of the pyramidal shape of the cutting edge, and it causes a more plowing action on the edges of the plies of the laminate. The mean percentage of increase in cutting force with tool T2 when compared to tool T1 at all three spindle speeds was calculated as 56.7% and mean percentage of increase in cutting force with tool T3 when compared to tool T1 at all three spindle speeds was calculated as 58.66%. The minimum force measured was 15.21 N at a spindle speed of 3000 rpm and feed of 0.1 mm/rev when machining with tool T1. The maximum force measured during machining was 59.26 N at a spindle speed of 9000 rpm and feed of 0.2 mm/rev when machining with fluted tool T3. The reason for this highest forces in tool T3 is that the tool cut the largest chip per tooth (three times as large as the other ones). The cutting force is one important process criteria for considering the surface damage of workpieces and failures in the cutting tool.

### 3.2. Surface Roughness

**Figure 6:**
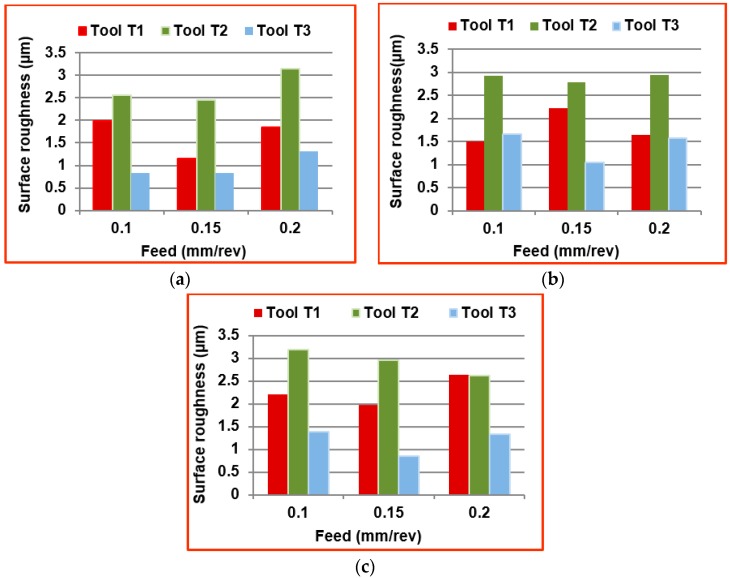
Comparison of surface roughness at different machining conditions with (**a**) 3000 rpm; (**b**) 6000 rpm; (**c**) 9000 rpm.

Figure 6a–c shows the surface roughness values measured at different spindle speeds and feeds with different kind of tools in the transverse direction (perpendicular to feed direction). The *Ra* values were measured using a surface roughness tester for a cut off length of 0.8 mm at three different positions on the machined edge of the workpiece. The measured values were averaged and considered for analysis. The tool T1 gives a moderate surface roughness due to the presence of trapezoidal cutting edge, provides better surface finish when compared to tools T2 and T3. The mean percentage decrease in surface roughness value with tool T1, when compared to T2, is calculated as 32.87%. The surface roughness obtained using tool T3 at all spindle speeds is less because of the reason that the tool T3 has cutting edges in the form of helical flutes. The mean percentage decrease in surface roughness value with tool T3, when compared to T2, is calculated as 57.89%. The scooping action of these flutes while machining results in lowering the roughness in the edges of the machined workpieces. The tool T2 gives the higher surface roughness value at all spindle speeds due to the presence of a sharp cutting edge of pyramid shaped tooth profile.

### 3.3. Delamination

**Figure 7:**
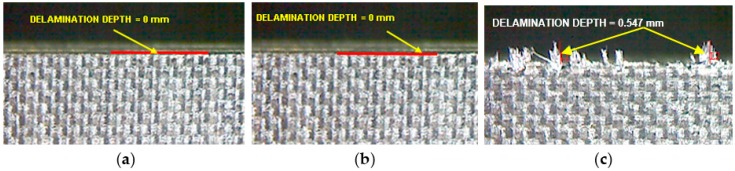
Microscopic images of edges of specimens with delamination depth measured after edge trimming with (**a**) Tool T1; (**b**) Tool T2 and (**c**) Tool T3.

Delamination is the measure of surface quality, and it was measured with a tool maker’s microscope. Figure 7a–c shows the delamination depth on specimens machined with different tools. It was observed that no delamination was present while using tool T1 and tool T2. Also, the axial force developed was small and thereby the delamination of top plies was also controlled to very minimum. The reason for this is due to the unique geometry of the cutting points on tools T1 and T2. There was significant delamination value measured as 0.547 mm while using tool T3 because the tool develops a high axial force and that in turn separates or disintegrates the extreme top plies. Another reason for delamination with the helical tool is that the chip per tooth is three times higher than the other tools as the number flutes in the helcal fluted tool is one-third of the number of flutes in both the router tools. Also, the chip is not broken into small segments because of the continuous cutting edge in the helical fluted tool. These continuous chips make the impact of the cutting edge on the laminate more severe to cause delamination.

### 3.4. AE Signal Measurement

**Figure 8:**
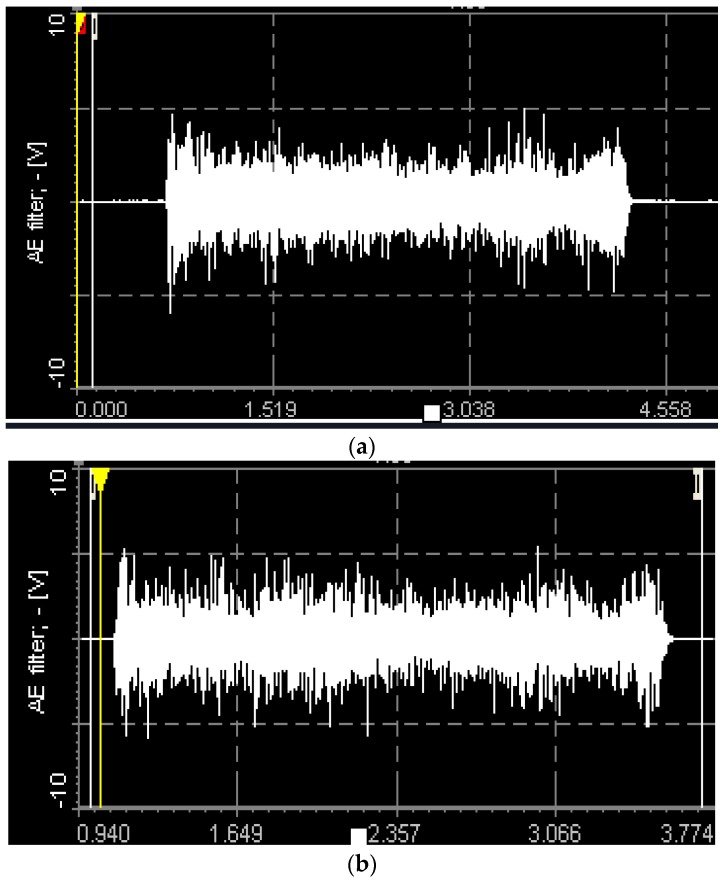

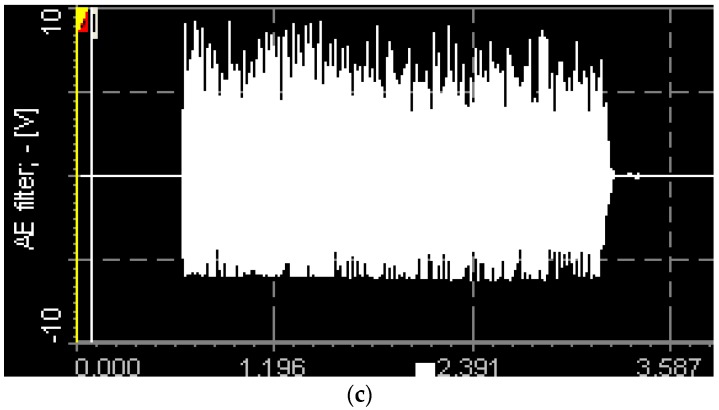
Time domain AE Out (Filter) signals recorded at cutting condition of 9000 rpm, 0.2 mm/rev, 0.5 mm doc (during 15th pass) using (**a**) Tool T1; (**b**) Tool T2; (**c**) Tool T3.

The AE signals were recorded and measured for the 15 passes (70 mm of each pass) equal to the length of machining of 1 m approximately at the cutting condition of high spindle speed of 9000 rpm and high feed of 0.2 mm/rev. AE Out (Filter) values are measured with a sampling rate of 2.5 MHz, and the mean values were used for the analysis. Figure 8a–c shows the images of AE signals recorded during machining at this condition with different tools.

The AE signal output values measured at the spindle speed of 9000 rpm and the feed of 0.2 mm/rev and the values are listed in Table 5.

The AE waves caused by cutting action between the cutting tool the workpiece can be considered as being included as part of the performance of the cutting tool. AE Out (Filter) mean value increases as the wear in the tool increases during continous machining. The magnitude of the AE signal can be considered as the measure of performance of the tool. The higher the AE signal, the poorer the performance of the tool.

**Figure 9:**
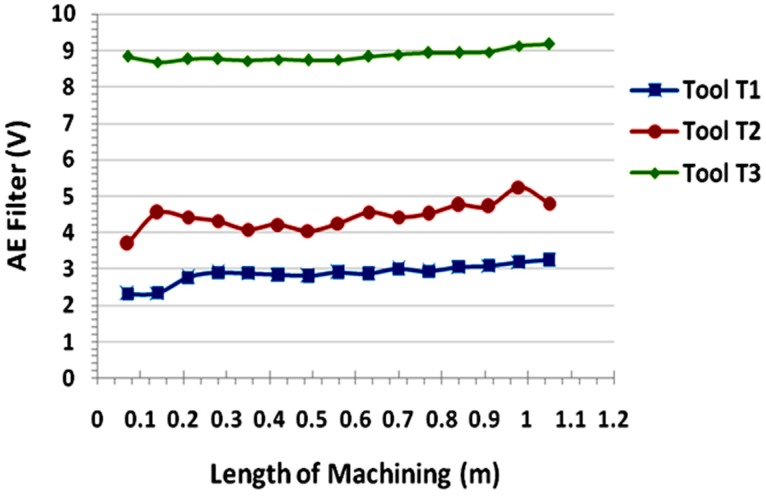
AE Out (filter) values Vs Length of machining for different tools.

From the Figure 9, it was observed that AE Out (Filter) value increases with the increase in the length of machining for all three types of tools. In other words, tool performance decreases as the length of machining progress. AE signal level is the function of machining performace. At pass 1, when there is no tool wear, the signal level of T3 is higher than T2 and T1. This is because of chip thickness and tool geometry. Since the router tool T1 had lowest chip thickness/tooth and better tool geometry when compared to tool T2 & T3, resulting in lowest signal values of AE out filter values. Therefore, the tool T1 can be considered as the tool with better cutting efficiency than tools T2 and T3. The fluted tool had highest AE out filter values that in turn indicates the poor cutting efficiency while trimming of CFRP.

Table 6 depicts the output responses such as cutting force, surface roughness, and delamination for the three different tools used. From the analysis it was concluded that tool T1 is generating low cutting force, no delamination with moderate surface roughness, and provides high cutting efficiency while trimming. Whereas the tool T3 is generating higher cutting force and high delamination with higher surface finish. The tool T2 is giving moderate cutting force, high surface roughness, and low delamination.

## 4. Mathematical Modeling

In this work, the experimental results corresponding to the effects of spindle speed and feed on resultant force of the CFRP have been investigated using regression analysis and Artificial Neural Network (ANN). The resultant cutting forces can be predicted by using regression and ANN techniques.

### 4.1. Regression Modeling

The regression equations were developed using Minitab software with the measured set of experimental data to predict the cutting force values at any combination of spindle speed and feed.

The resultant cutting force equation for the tool T1 was obtained as

*Resultant cutting force for T1* = 2.70 + (0.00105 × *spindle speed*) + (83.0 × *feed*) (2)

The value of the correlation coefficient *R*^2^ = 82.5%. 

The resultant cutting force equation for the tool T2 was obtained as

*Resultant cutting force for T2* = 32.3 + (0.00150 × *spindle speed*) + (51.6 × *feed*) (3)

The value of the correlation coefficient *R*^2^ = 88.7%.

The resultant cutting force equation for the tool T3 was obtained as

*Resultant cutting force for T3* = 35.9 + (0.00151 × *spindle speed*) + (44.5 × *feed*) (4)

The value of the correlation coefficient *R*^2^ = 93.9%.

### 4.2. Artificial Neural Network (ANN)

ANN provides linear and nonlinear modeling without the requirement of preliminary information and assumption as to the relationship between input and output variables. The traditional theoretical model could not include the influence of all the parameters considered and thereby results in a large percentage of error. Also, the relationship between the parameters themselves, being complex, could not be modeled with the theoretical relationship. These limitations of the theoretical model have been overcome by the usage of a neural network.

**Figure 10:**
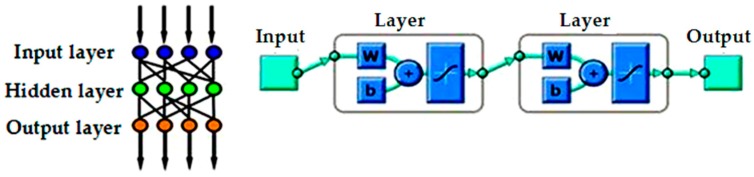
Feed Forward Network Structure.

The neural network is an information processing system in which the process is carried out using elements called neurons that are interconnected by a link. The concept is based on ideal neuron which is assumed to be responding optimally to applied inputs. The links possess associated weights which are multiplied with the input signal to produce output. There are three layers such as input, hidden, and output as shown in Figure 10.

A neural network model has been generated using MATLAB 2013, and it is trained to predict the velocity with the given input parameters. In a feed forward neural network, the units do not form a directed cycle. The information moves in only one direction—forward—from the input nodes, through the hidden nodes and to the output nodes. The training process adjusts the connection weight and bias of network to minimize the error function. The adjustment of connection weight is conducted by back propagating the errors to the network. The neural network has been developed using neural network toolbox in MATLAB 2013, parameters for developing ANN model is shown in Table 7.

The spindle speed and feed were taken as input vectors, and resultant forces were taken as target vectors. The neurons were trained by the incremental value of 10. The number of neurons was selected such that the value of *R* (correlation between outputs and targets) is almost one.

### 4.3. Comparison between Measured Values with ANN and Regression Modeling

The predicted values of cutting forces for different cutting conditions for the different tool geometries were found and compared between the regression method and ANN.

From the results shown in Table 8, Table 9 and Table 10, it was observed that prediction of cutting forces through Artificial Neural Network model yielded better results when compared to regression model.

**Figure 11:**
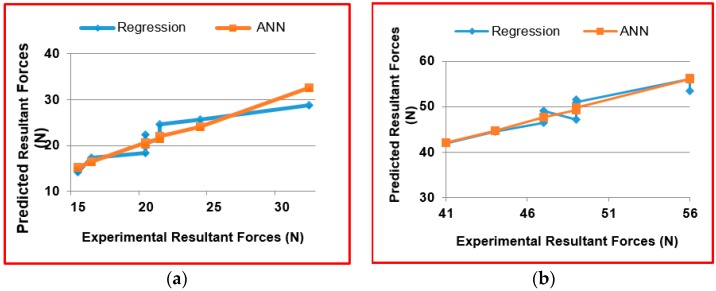

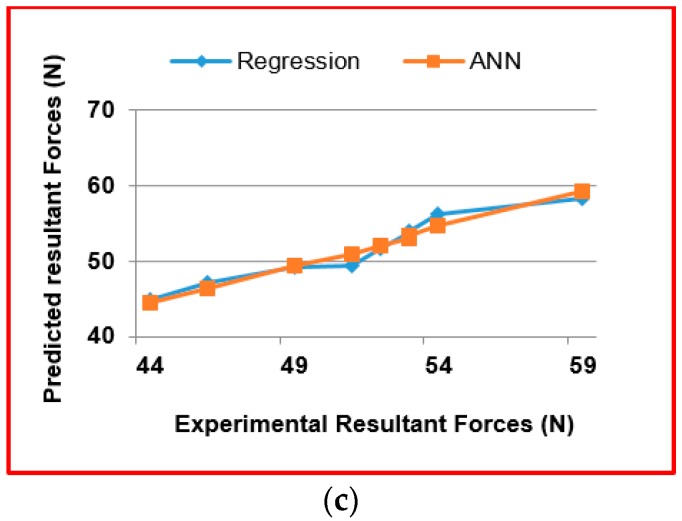
Comparison between regression model and ANN model with the experimental resultant forces. (**a**) at Tool T1; (**b**) at Tool T2; (**c**) at Tool T3.

From Figure 11, it was observed that the ANN model can predict better values when to compared to the regression model.

## 5. Conclusions

This experimental investigation was carried out to investigate the machining characteristics of CFRP using router tools and helical-fluted end mill during high-speed edge trimming.

- It was found that the tool T1 generated lower cutting force and moderate surface roughness with no delamination while machining of CFRP materials. The performance of the tool was also found to be the best among the three tools as the cutting tooth of trapezoidal shape with more cutting area creates lower surface damages in the trimmed edges.
- It was observed that the machined edges of CFRP specimens have no delamination as tool T1 and T2 have discontinuous cutting edges unlike in the case of continuous edge in the helical fluted tool.
- The tool T2 generated higher cutting force and surface roughness when compared to the other two types of tools. The cutting tooth has a small flat edged pyramid form creates more indentations on the workpiece surface. That, in turn, results in increasing the surface roughness.
- The tool T3 generated higher cutting forces and more delamination when compared to T1 and T2. The continuous flutes with higher helical angle cause the pulling action of the extreme top and bottom plies of the laminate which results in delamination [14].
- The regression equations and ANN models were developed to predict the cutting force values for any combination of spindle speed and feed. The predicted values of cutting forces obtained through ANN are accurate when compared to regression analysis.

## Figures and Tables

**Table 1:** Cutting tools used and their specification.

Tool	Specification	Image of the Tool	Tooth Shape	Tooth Profile
Tool T1	Pitch = 3.42 mm	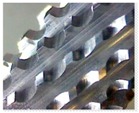	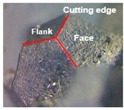	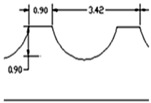
	No. of flutes—12			
	Tooth Shape—Trapezoidal			
Tool T2	Pitch = 2.42 mm	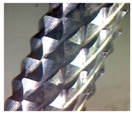	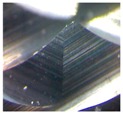	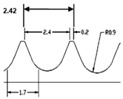
	No. of flutes—12			
	Tooth Shape—Pyramidal			
Tool T3	Helix angle 30°	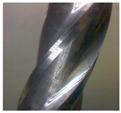	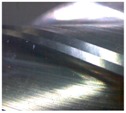	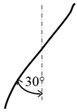
	No. of. flutes—4			
	Tooth Shape—Helical fluted			

**Table 2:** Specification of Makino S33 VMC.

Description	Specifications
Axes travel X, Y, Z	650 mm× 500 mm × 450 mm
Rapid Transverse XYZ	40 m/min
Axis feed rate	1–40,000 mm/min
Spindle speed range	200–20,000 rpm
Accuracy	0.003 micron
Repeatability	0.002 micron

**Table 3:** Machining Conditions.

Serial No.	Cutting Parameters	Levels
1	Spindle speed (rpm)	3000, 6000, 9000
2	Feed (mm/rev)	0.1, 0.15, 0.2
3	Radial depth of cut (mm)	0.5

**Table 4:** Resultant cutting forces and surface roughness values.

Spindle Speed (rpm)	Feed (mm/rev)	Resultant Cutting Force (N)			Surface Roughness *Ra* (µm)		
		T1	T2	T3	T1	T2	T3
3000	0.1	15.213	41.850	44.088	1.996	2.565	0.830
3000	0.15	20.412	44.190	46.288	1.153	2.459	0.828
3000	0.2	20.549	49.006	49.803	1.845	3.141	1.313
6000	0.1	16.501	47.400	51.442	1.499	2.931	1.655
6000	0.15	21.315	47.462	52.330	2.226	2.780	1.045
6000	0.2	24.012	49.523	53.043	1.642	2.946	1.579
9000	0.1	20.570	49.801	53.219	2.213	3.192	1.391
9000	0.15	21.913	56.325	54.818	1.973	2.955	0.844
9000	0.2	32.614	59.265	56.004	2.629	2.615	1.329

**Table 5:** AE signal Out (Filter) inVolts for different passes.

No. of Passes	AE Signal Out (Filter) inVolts		
	Tool T1 (Volts)	Tool T2 (Volts)	Tool T3 (Volts)
1	2.32	3.69	8.84
2	2.35	4.54	8.68
3	2.77	4.43	8.76
4	2.90	4.33	8.77
5	2.89	4.08	8.72
6	2.85	4.23	8.75
7	2.81	4.05	8.73
8	2.91	4.26	8.74
9	2.88	4.56	8.83
10	3.01	4.42	8.88
11	2.94	4.51	8.93
12	3.07	4.75	8.93
13	3.10	4.71	8.95
14	3.20	5.25	9.12
15	3.26	4.81	9.18

**Table 6:** Consolidated results.

Tool	Output Responses			
	Cutting Force	Surface Roughness	Delamination	Cutting Efficiency
Tool T1	Low	Moderate	Low	High
Tool T2	Moderate	High	Low	Moderate
Tool T3	High	Low	High	Low

**Table 7:** Parameters for developing ANN Model.

Structure	Feed Forward
Algorithm	Back propagation
Type of Training	Trainlm
Transfer function	TANSIG
Number of iterations	1000 (max epoch)

**Table 8:** Predicted cutting forces using regression model and ANN model for tool T1.

Spindle Speed (rpm)	Feed (mm/rev)	Actual Force (N)	Force in Regression (N)	Error in Regression (%)	Force in ANN (N)	Error in ANN (%)
3000	0.1	15.21	14.15	6.96	15.21	0.03
3000	0.15	20.41	18.30	10.38	20.56	0.73
3000	0.2	20.55	22.45	9.24	20.30	1.20
6000	0.1	16.50	17.30	4.84	16.41	0.50
6000	0.15	21.32	21.45	0.61	21.56	1.12
6000	0.2	24.01	25.60	6.62	24.10	0.37
9000	0.1	20.57	20.45	0.58	20.63	0.29
9000	0.15	21.91	24.60	12.27	21.98	0.31
9000	0.2	32.61	28.75	11.83	32.59	0.03

**Table 9:** Predicted cutting forces using regression model and ANN model for tool T2.

Spindle Speed (rpm)	Feed (mm/rev)	Actual Force (N)	Force in Regression (N)	Error in Regression (%)	Force in ANN (N)	Error in ANN (%)
3000	0.1	41.85	41.96	0.26	42.19	0.81
3000	0.15	44.19	44.54	0.79	44.76	1.28
3000	0.2	49.01	47.12	3.85	49.23	0.44
6000	0.1	47.40	46.46	1.98	47.80	0.84
6000	0.15	47.46	49.04	3.32	47.73	0.58
6000	0.2	49.52	51.62	4.24	49.51	0.00
9000	0.1	49.80	50.96	2.32	49.79	0.00
9000	0.15	56.33	53.54	4.95	56.32	0.00
9000	0.2	56.01	56.12	0.19	56.13	0.21

**Table 10:** Predicted cutting forces using regression model and ANN model for tool T3.

Spindle Speed (rpm)	Feed (mm/rev)	Actual Force (N)	Force in Regression (N)	Error in Regression (%)	Force in ANN (N)	Error in ANN (%)
3000	0.1	44.08	44.88	1.81	44.49	0.94
3000	0.15	46.28	47.11	1.78	46.43	0.33
3000	0.2	49.80	49.33	0.94	49.50	0.60
6000	0.1	51.44	49.41	3.94	50.96	0.93
6000	0.15	52.33	51.64	1.32	52.13	0.37
6000	0.2	53.04	53.86	1.54	52.99	0.09
9000	0.1	53.22	53.94	1.35	53.36	0.26
9000	0.15	54.82	56.17	2.45	54.82	0.00
9000	0.2	59.26	58.39	1.46	59.21	0.08
